# Why Does the Molecular Structure of Broadly Neutralizing Monoclonal Antibodies Isolated from Individuals Infected with HIV-1 not Inform the Rational Design of an HIV-1 Vaccine?

**DOI:** 10.3934/publichealth.2015.2.183

**Published:** 2015-05-06

**Authors:** Marc H V Van Regenmortel

**Affiliations:** CNRS, UMR7242 – Institut de Recherche de l'Ecole de Biotechnologie de Strasbourg (IREBS), Université de Strasbourg, Illkirch 67400, France Email: vanregen@unistra.fr; Tel: +27-793376766

**Keywords:** antibody polyspecificity, Darwinian natural selection, design metaphor, discontinuous HIV epitopes, rational HIV-1 vaccine design, reverse vaccinology

## Abstract

It is commonly assumed that neutralizing Mabs that bind to the HIV-1 Env glycoprotein are more specific reagents than anti-HIV-1 polyclonal antisera and that knowledge of the structure of these Mabs facilitates the rational design of effective HIV-1 vaccine immunogens. However, after more than ten years of unsuccessful experimentation using the structure-based reverse vaccinology approach, it is now evident that it is not possible to infer from the structure of neutralizing Mabs which HIV immunogens induced their formation nor which vaccine immunogens will elicit similar Abs in an immunized host. The use of Mabs for developing an HIV-1 vaccine was counterproductive because it overlooked the fact that the apparent specificity of a Mab very much depends on the selection procedure used to obtain it and also did not take into account that an antibody is never monospecific for a single epitope but is always polyspecific for many epitopes. When the rationale of the proponents of the unsuccessful rational design strategy is analyzed, it appears that investigators who claim they are designing a vaccine immunogen are only improving the binding reactivity of a single epitope-paratope pair and are not actually designing an immunogen able to generate protective antibodies. The task of a designer consists in imagining what type of immunogen is likely to elicit a protective immune response but in the absence of knowledge regarding which features of the immune system are responsible for producing a functional neutralizing activity in antibodies, it is not feasible to intentionally optimize a potential immunogen candidate in order to obtain the desired outcome. The only available option is actually to test possible solutions by trial-and-error experiments until the preset goal is perhaps attained. Rational design and empirical approaches in HIV vaccine research should thus not be opposed as alternative options since empirical testing is an integral part of a so-called design strategy.

## 1. Introduction

It has been suggested that our inability over the past 25 years to develop an effective HIV vaccine is partly due to the fact that investigators adhered to several unwarranted assumptions and paradigms that made them pursue unfruitful research strategies [1],[2]. One such misleading assumption central to the structure-based reverse vaccinology approach [3] was the belief that when an HIV-1 Env epitope is found to bind to a broadly neutralizing monoclonal antibody (bnMab), this epitope should also be able to induce similar neutralizing antibodies when used as an immunogen [4]. A related assumption was that HIV-1 Env epitopes, targeted by hypermutated bnMabs that are produced in HIV-1 infected individuals after a lengthy process of antibody affinity maturation, would be able to trigger a protective immune response in naive individuals [5],[6].

The present review will discuss another detrimental assumption that impeded progress in the HIV vaccine field, namely the belief that a Mab that binds to the HIV-1 Env protein is a more appropriate and specific reagent for studying HIV immunology and vaccine immunogenicity than a polyclonal anti-HIV antiserum.

Such a belief arises when antibodies are perceived to be monospecific for a single epitope rather than polyspecific for a number of related or unrelated epitopes. In an antiserum containing antibodies directed to different epitopes of a multi-epitopic viral antigen, each individual antibody will also cross-react with numerous epitopes present in other antigens. However, since these cross-reactive epitopes will be different for each type of antibody found in the antiserum, the cross-reactions will be diluted out in the antiserum and may not be apparent. In contrast, the cross-reactions of a single Mab will not be masked in this manner, and the Mab may therefore appear to be less specific than the antiserum. A polyclonal antiserum will thus have a greater collective specificity for a multi-epitopic viral antigen than a Mab since it contains many antibodies, directed to several different viral epitopes, that give rise to an additive specificity effect [7],[8]. The presence of such antibodies in the antiserum often also produces a beneficial, protective neutralization synergy. Most protective immune responses against pathogens are polyclonal and involve the collective neutralizing activities of antibodies directed to separate epitopes. When one antibody present in an anti-HIV antiserum binds to the Env glycoprotein, it may induce a conformational change in the protein and this could then allow another antibody in the antiserum to bind to a newly exposed epitope in Env which could lead to neutralization synergy [9],[10]. When an antiserum contains certain combinations of antibodies directed to separate epitopes of a virus, antibody synergy may achieve a higher degree of neutralization than would arise from the simple additive effect of any two randomly chosen neutralizing antibodies [11],[12]. It is now generally accepted that the most effective vaccine-induced antibody response will always be polyclonal and directed to multiple neutralizing epitopes [13].

Early attempts at developing an HIV vaccine were based either on humoral immunity, on cell-mediated immunity or a combination of both [1]. More recently, it became accepted that a vaccine eliciting neutralizing antibodies was likely to be adequate to confer protection against HIV infection and the present review will only discuss that particular vaccine strategy. Most successful viral vaccines act by mimicking the protective antibody immune response that is usually generated when a virus causes a natural infection in a host. However, during HIV infection, most individuals do not develop protective antibodies able to control viral replication. This means that a successful HIV vaccine must be able to achieve something that the human immune system is normally not able to do when it encounters the virus. This is probably one of the reasons why attempts to develop a preventive HIV vaccine have failed so far.

It has been claimed [14] that if we understood why the human immune system is mostly unable to elicit antibodies that protect against HIV infection, this would facilitate the ultimate development of an effective vaccine. The rationale for such optimism is not obvious since evidence for the absence of protection in one case may throw little light on the absence of evidence for protection in other cases. There is, in fact, no ground for claiming that if we understood why most anti-HIV Mabs are not protective, this would necessarily improve our ability to develop a vaccine able to elicit neutralizing antibodies. A related unwarranted claim is the assertion that if we study the epitope targets of large numbers of bnMabs, this will give us useful knowledge that will "inform" rational vaccine design [14]. The aim of this review is to analyse the validity of such claims since their acceptance may lead vaccinologists to pursue unfruitful lines of investigation.

## 2. The antibody response to the HIV-1 envelope glycoprotein complex (Env)

The HIV-1 Env glycoprotein consists of about 15 spikes per virion which are embedded in the viral membrane [15]. It has been suggested that this low number of spikes makes it difficult to achieve bivalent antibody binding to two adjacent spikes and that this could be responsible for the high degree of polyreactivity and self reactivity of anti-HIV-1 bnMabs and for the fact that these antibodies require a lengthy process of affinity maturation to acquire neutralizing activity [15]–[17]. Each spike is made up of trimers of three gp120 surface glycoproteins that are non-covalently attached to three gp41 transmembrane glycoproteins. Besides functional glycoprotein trimers, the viral membrane also displays gp120-gp41 monomers and single gp41 molecules as well as other non-functional fragments [18],[19]. The structure of the spikes has been analyzed by cryo-electron microscopy using Env bound to Mabs as well as unliganded Env [20],[21].

Most gp120 epitopes are structurally discontinuous and many of them are so-called neotopes [22] that arise from the native quaternary structure that is present only in intact, functional spikes [23]. Neotopes arise either from the juxtaposition of residues in neighbouring protein subunits that are recognized by antibodies as a single epitope, or from conformational changes induced in the proteins by intersubunit interactions [24].

Many anti-Env bnMabs target conserved protein regions such as the CD4 receptor binding site and the transiently exposed membrane -proximal external region (MPER) located at the base of the spike's stem [25]. Such antibodies inhibit HIV infection by either blocking binding to cellular receptors or by interfering with the fusion of viral and cellular membranes. Both these activities must be preserved if the virus is to maintain fitness [26],[27].

It is commonly accepted that the most effective way of dissecting the antibody response to HIV infection consists in using neutralizing Mabs derived from infected individuals. In recent years, new antibody screening and selection technologies have made it possible to isolate many additional neutralizing Mabs [27]–[29] and this has reinforced the expectation that if the structure of these antibodies was elucidated, it would facilitate the design of vaccine immunogens able to elicit a neutralizing immune response [30]. There is considerable interest in these Mabs because numerous immunotherapy studies have demonstrated that they are able to provide sterilizing protection *in vivo* [11],[31]. However, the isolation of Mabs useful for passive immunotherapy does not improve our ability to discover which vaccine immunogens will elicit such antibodies by active immunization [6]. The structure of a neutralizing, protective Mab, unfortunately does not in any way tell us which immunogen induced its formation nor which vaccine immunogen would be able to elicit similar neutralizing antibodies in an immunized host.

The difficulties one encounters when trying to make use of the known structure of certain bnMabs for developing effective HIV-1 vaccine immunogens are well illustrated in the case of the many anti-HIV-1 bnMabs found to possess very long CDRH3 regions of 20-34 residues compared to the average length of 16 residues of this region usually found in human B cells [32]. These long CDRH3 regions protrude from the paratope surface [33] and can penetrate the glycan shield of the Env trimer to interact with gp120 loops or reach conserved residues on gp41. It was initially believed that knowledge of the immunological mechanisms responsible for the generation of bnMabs with long CDRH3 sequences would help the design of an effective vaccine but this was found not to be the case. Long CDRH3 sequences are present in the mature naïve B cell repertoire and arise during recombination of VDJ gene segments before the antigen-driven process of antibody affinity maturation [34]. Long CDRH3 sequences can also arise from VH replacement that occurs when a short stretch of nucleotides from previously rearranged VH genes are left within newly formed CDRH3s. However, knowledge of these mechanisms did not make it possible to increase the generation of bnAbs endowed with long CDRH3s [32].

It is a reductionist fallacy to believe that the biological, neutralizing activity of an antibody which involves a context-dependent ternary interaction between antibody, virus and host can be reduced to and explained by the physico-chemical structure of the participating antibody molecules [6]. Chemical antigenicity is confused with biological immunogenicity when it is assumed that the immunogenic, protective potential of the Env glycoprotein can be deduced and predicted from its antigenic properties.

During the immune response to HIV-1, neutralizing antibodies are not preferentially selected compared with non-neutralizing antibodies [27]. B cell receptors are actually selected for their capacity to capture viral antigens and this selection process is blind to the presence of virus neutralizing activity in the antibodies that will subsequently be secreted by plasmocytes. In fact, it seems that individuals from whom nAbs are isolated do not benefit from the presence of these antibodies for controlling viral replication, possibly because only a small fraction of virus-specific memory B cells will be quickly reactivated and will differentiate into plasmablasts [27]. Furthermore, since bnAb responses typically appear only two to three years after infection, the kinetics of the evolving B cell response also lag behind the rapidly diversifying virus, preventing the antibodies from controlling the infection [35]. The Env-directed antibody response is believed to reflect the continual stimulation by evolving antigenic variants of Env rather than the continued production of antibodies elicited by the Env protein of the originally infecting virus [36] When the extent of change in bnAb susceptibility of HIV-1 within individual progressors was studied during the course of infection, it was found that viral variants resistant to one or more bnAbs could develop in most individuals [37]. This led to the suggestion that since viral resistance against bnAb- mediated neutralization generally developed when autologous serum neutralization had faded, these changes were unlikely to have been driven by escape from autologous humoral immunity [37]. The relevance of these findings to vaccine development is unclear since protection against HIV infection requires high titers of nAbs at mucosal sites that are seldom obtained during chronic infection or by Env immunization [36].

## 3. Dissecting the antigenic structure of HIV-1 with Mabs has unforeseen consequences

In spite of their usefulness for dissecting immune responses to proteins, the use of Mabs introduced a distorted view of the antigenic surface of proteins because it encouraged investigators to concentrate on small numbers of single, discrete epitopes recognized by individual Mabs that were considered to be potential vaccine immunogens. As a result, researchers focused on artificial boundaries between the numerous overlapping epitopes present on the surface of viral proteins and disregarded the fact that a protein surface is an antigenic and functional continuum consisting of a large number of epitopes [38]–[40].

The use of Mabs introduced another bias because the apparent specificity of a Mab very much depends on the selection process that was used to obtain it [41]. For instance, Mabs are often selected for their ability to bind to individual linear peptides in a peptide library that are believed to mimic a viral epitope. It should then come as no surprise that a Mab selected in this way will tend to bind more strongly to a peptide than to the virions used for eliciting the Mabs. When a bnMab binds to a short region of a virus protein, this could simply reflect the fact that the Mab was selected for its ability to bind to that peptide in an immunoassay. When short peptide regions of the HIV-1 gp41 MPER are found to bind to bnMabs, there is thus no reason to believe that these Mabs were induced by the peptides they react with and not by chemically heterogeneous, more complex or transient epitopes present in gp41 [6],[42].

When a bnMab is found by X-ray crystallography of a Mab- Env complex to bind to a discontinuous epitope, it is not possible to isolate the epitope in its active form by extracting it from the native protein in order to show that it possesses binding activity on its own [41]. Since a discontinuous epitope possesses binding and immunogenic activity only when it is embedded in a protein, its immunogenicity can only be tested by using the native protein as immunogen. This inevitably produces a heterogeneous response against the numerous epitopes present in the protein and makes it impossible to derive any useful information regarding the separate immunogenicity of the discontinuous epitope of known crystallographic structure [42]. Neutralizing Mabs are thus useful for analyzing the antigenicity of HIV-1 proteins but not for studying the immunogenicity of the discontinuous epitopes they recognize.

Another limitation inherent in the use of Mabs for helping to develop an HIV-1 vaccine arises from the polyspecificity of antibody molecules [4],[8],[43]. In view of the degenerate nature of the immune system, there is no single intrinsically specific epitope for any Ab but only a diverse group of potential epitopes able to bind to it with various degrees of fit [44]–[47]. Since the epitope structure determined by the crystallographic analysis of a bnMab- HIV Env complex corresponds to only one of the many epitopes that could be accommodated by that Mab, there is no reason why the epitope identified in the complex should indicate which immunogen induced the Mab, thereby making it an hypothetical vaccine candidate.

## 4. What does it mean to rationally design an HIV-1 vaccine ?

Proponents of the rational design approach to HIV-1 vaccine development maintain that when they have determined the structure of a bnMab and its HIV-1 Env epitope, this information should allow them to design a vaccine immunogen that will elicit antibodies able to neutralize the virus. The rational design of an HIV-1 vaccine has been advocated for many years [14],[48]–[52] and its adepts do not seem to be disheartened by the persistent failure of that approach to develop an HIV-1 vaccine. It may be instructive, therefore, to analyze the rationale of those who remain committed to such an unsuccessful strategy. This is best done by clarifying what vaccinologists actually mean when they claim that they are engaged in “rationally designing a vaccine”.

Doing something by design is doing it intentionally. For instance, adepts of so-called “intelligent design” argue that a mythical, intelligent deity is responsible for designing all living forms on our planet and they pretend to explain the occurrence of life and evolution by the creationist credo: “God did it” [53]. Such a belief is today rejected by most scientists because they no longer accept that living organisms and the biological functions they exhibit were designed by the preconceived plan of a designer rather than being shaped by the filter and pressure of Darwinian natural selection. It has been argued elsewhere [53] that design terminology is inappropriate both for explaining the appearance of biological organisms on earth and for describing the activities of vaccinologists when they empirically select a vaccine immunogen able to elicit antibodies endowed with the rather complex biological function of HIV-1 neutralization.

The term design refers to the deliberate conceiving of an articial, novel object or process by an intelligent being. In the case of a vaccine immunogen, it describes the structure-based strategy used in rational drug design [54] when it is applied to the improvement of the binding complementarity between an antigen and an antibody in the narrow context of a single Mab-epitope pair. In this case, it is indeed justified to refer to the rational design of the HIV-1 Env glycoprotein with respect to its ability to bind a single bnMab molecule [55] or to the rational design of one HIV antibody molecule with respect to one HIV epitope [56]. In both cases, it is one epitope or one paratope that is being designed to achieve improved binding complementarity and the authors in fact only study the antigen binding capacity or antigenicity of a viral protein and not its immunogenicity. The claim that what is being rationally designed is actually an HIV-1 vaccine immunogen [14] is based on the misconception that improving the binding capacity of one viral epitope with respect to a single Mab amounts to designing an immunogen capable of generating protective antibodies. This confusion is due to the assumption that studying the binding capacity of bnMabs can reveal which HIV-1 immunogens are able to elicit neutralizing antibodies. It is ultimately the consequence of confounding antigenicity and immunogenicity and of assuming that if an HIV-1 epitope reacts with a bnMab, it should also be able to induce similar, protective antibodies in an immunized host. In the case of HIV, the enormous antigenic variability of the virus and the requirement for extensive antibody affinity maturation to obtain neutralizing antibodies invalidates such an approach that may however work with other viruses or antigens. On the other hand, the positive results obtained when rational vaccine design was used with respiratory syncytial virus [57] may not be a valid proof of concept for what can be expected to happen in the case of HIV-1.

It is actually not feasible to rationally design an HIV-1 vaccine immunogen because the designer would need to know beforehand which features and components of the human immune system are responsible for the induction of a protective immune response. Since such knowledge is not available, it is impossible for a vaccinologist to conceive what type of immunogen is capable of eliciting protective antibodies and how its structure must be intentionally optimized in order to bring about the desired outcome. What is possible, of course, is to investigate the numerous factors, external to epitope-paratope recognition, that originate in the immunized host and control which antibodies will be produced by its immune system. This would require studying the host immunoglobulin repertoire, the presence of appropriate B cell receptors and cytokines, the occurrence of antibody affinity maturation and various cellular and regulatory mechanisms present only in the context of a given immune system [41]. Such studies require trial-and-error experimentation involving characteristics of the host and they fall outside the framework of a rational design strategy taken as a purely molecular engineering project for constructing effective vaccine immunogen molecules solely on the basis of the antigenic properties of the virus.

The task of a designer is to pose and solve an inverse problem, namely to imagine, using available knowledge, what would bring a desired outcome [58]. Various possible solutions can then be tested by trial and error experimentation until the preset goal is attained. It is thus misleading to oppose structure-based rational design and empirical approaches in HIV-1 vaccine research as if design is better science than empiricism [59]. Trial-and-error testing is entirely rational and forms an integral part of any attempt to develop an effective HIV-1 vaccine [41].

## 5. Conclusions

The structure-based rational design of an HIV-1 vaccine is based on the assumption that knowledge of the structure of vulnerable Env epitopes targeted by bnMabs will lead to the discovery of effective HIV-1 vaccine immunogens [4],[14],[41],[42]. After many years of unsuccessful experimentation by numerous laboratories, Mascola and Haynes in 2013 concluded that “our best attempts to construct vaccine immunogens that present these key epitopes to the immune system have failed to generate antibodies that neutralize most strains of HIV-1” and they acknowledged that “a structure-based approach in and of itself will likely not solve the HIV-1 vaccine problem” [13].

The unsuccessful RV paradigm utilizes the deceptive metaphor of a rational design terminology which is as inappropriate for designing improved vaccine immunogens as it is for attributing Darwinian natural selection to the activity of a designer involved in developing useful biological functions [53]. The RV paradigm has lost its popularity with HIV vaccinologists who no longer believe that HIV-1 epitopes of known structure can be directly transformed into effective vaccine immunogens. A new paradigm has replaced it which stresses that vaccine immunogenicity depends mainly on the biology of the immunized host. This paradigm advocates the study of bnAb lineages in infected individuals and their development from early infection through the process of antibody maturation to eventual potent neutralization activity. The aim is to drive the human immune response towards highly mutated anti-HIV-1 bnAbs by using sequential immunizations with various Env immunogens. Several teams have successfully analyzed the paths along which anti-HIV-1 bnAbs develop [58]–[66] but it is not clear yet whether unravelling large numbers of different antibody maturation pathways [67] will make it possible to identify a series of effective immunogens suitable for vaccinating large human populations.
